# Elderly versus younger patients with hereditary angioedema type I/II: patient characteristics and safety analysis from the Icatibant Outcome Survey

**DOI:** 10.1186/s13601-019-0272-9

**Published:** 2019-07-19

**Authors:** Anette Bygum, Teresa Caballero, Anete S. Grumach, Hilary J. Longhurst, Laurence Bouillet, Werner Aberer, Andrea Zanichelli, Jaco Botha, Irmgard Andresen, Marcus Maurer, for the IOS Study Group, W. Aberer, A. S. Grumach, R. Hakl, A. Bygum, C. Blanchard Delaunay, I. Boccon-Gibod, L. Bouillet, B. Coppere, A. Du Thanh, C. Dzviga, O. Fain, B. Goichot, A. Gompel, S. Guez, P. Y. Jeandel, G. Kanny, D. Launay, H. Maillard, L. Martin, A. Masseau, Y. Ollivier, A. Sobel, E. Aygören-Pürsün, M. Baş, M. Bauer, K. Bork, J. Greve, M. Magerl, I. Martinez-Saguer, M. Maurer, U. Strassen, E. Papadopoulou-Alataki, F. Psarros, Y. Graif, S. Kivity, A. Reshef, E. Toubi, F. Arcoleo, M. Bova, M. Cicardi, P. Manconi, G. Marone, V. Montinaro, M. Triggiani, A. Zanichelli, M. L. Baeza, T. Caballero, R. Cabañas, M. Guilarte, D. Hernandez, C. Hernando de Larramendi, R. Lleonart, T. Lobera, L. Marqués, B. Saenz de San Pedro, J. Björkander, C. Bethune, T. Garcez, H. J. Longhurst

**Affiliations:** 1 Department of Dermatology and Allergy Centre, Odense University Hospital, J.B. Winsløws Vej 4, Indgang 142, 5000 Odense C, Denmark; 2 Department of Clinical Research, University of Southern Denmark, Odense, Denmark; 3 Allergy Department, Hospital La Paz Institute for Health Research (IdiPaz), Biomedical Research Network on Rare Diseases (CIBERER, U754), Madrid, Spain; 4 Faculdade de Medicina ABC, Santo Andre, SP Brazil; 5 Department of Clinical Biochemistry and Immunology, Addenbrooke’s Hospital, Cambridge University Hospitals NHS Foundation Trust, Cambridge, UK; 6 National Reference Centre for Angioedema, Internal Medicine Department, Grenoble University Hospital, Grenoble, France; 7 Department of Dermatology and Venereology, Medical University of Graz, Graz, Austria; 8 Department of Biomedical and Clinical Sciences Luigi Sacco, University of Milan, ASST Fatebenefratelli Sacco, Milan, Italy; 9 Shire, a Takeda company, Zug, Switzerland; 10 Department of Dermatology and Allergy, Dermatological Allergology, Charité – Universitätsmedizin Berlin, Berlin, Germany

**Keywords:** Hereditary angioedema, Icatibant Outcome Survey, Safety, Elderly

## Abstract

**Background:**

Hereditary angioedema with C1 inhibitor deficiency (C1-INH-HAE) is characterized by recurrent swelling in subcutaneous or submucosal tissues. Symptoms often begin by age 5–11 years and worsen during puberty, but attacks can occur at any age and recur throughout life. Disease course in elderly patients is rarely reported.

**Methods:**

The Icatibant Outcome Survey (IOS) is an observational study evaluating the safety, tolerability, and efficacy of icatibant. We conducted descriptive analyses in younger (age < 65 years) versus elderly patients (age ≥ 65 years). Here, we report patient characteristics and safety-related findings.

**Results:**

As of February 2018, 872 patients with C1-INH-HAE type I/II were enrolled, of whom 100 (11.5%) were ≥ 65 years old. Significant differences between elderly versus younger patients, respectively, were noted for median age at symptom onset (17.0 vs 12.0 years), age at diagnosis (41.0 vs 19.4 years), and delay between symptom onset and diagnosis (23.9 vs 4.8 years) (P ≤ 0.0001 for all). Median age at diagnosis was significantly higher in elderly patients regardless of family history (P < 0.0001). Throughout the study, icatibant was used to treat 6798 attacks in 574 patients, with 63 elderly patients reporting 715 (10.5%) of the icatibant-treated attacks. No serious adverse events (SAEs) in elderly patients were judged to be possibly related to icatibant, whereas two younger patients reported three possibly related SAEs. Excluding off-label use and pregnancy (evaluated for regulatory purposes), the percentage of patients with at least one possibly/probably related AE was similar for elderly (2.0%) versus younger patients (2.7%). No deaths linked to icatibant treatment were identified. All related events in elderly patients were attributed to general disorders/administration site conditions, whereas related events in younger patients occurred across various system organ class designations.

**Conclusions:**

Elderly patients with C1-INH-HAE were significantly older at diagnosis and had greater delay in diagnosis than younger patients. Elderly patients contributed to approximately 10% of the icatibant-treated attacks. Our analysis found similar AE rates (overall and possibly/probably related) in icatibant-treated elderly versus younger patients, despite the fact that elderly patients had significantly more comorbidities and were receiving a greater number of concomitant medications. Our analysis did not identify any new or unexpected safety concerns.

## Background

Hereditary angioedema with C1 inhibitor deficiency (C1-INH-HAE) is a rare, chronic disease caused by *SERPING1* gene mutations [1]. Clinical manifestations include recurrent, unpredictable episodes of bradykinin-mediated swelling in subcutaneous or submucosal tissues that are associated with a heavy burden of illness [2, 3]. Attacks can range from mild to severe and debilitating [4], negatively affecting patients’ ability to attend school, be productive at work, or participate in daily social activities [5].

Onset of symptoms typically begins within the first two decades of life (often by age 10–11 years), with intensity of attacks worsening during puberty [6]. However, C1-INH-HAE attacks may occur at any age and recur throughout a patient’s lifetime [7, 8]. As such, treatment of acute attacks is a lifelong necessity and can pose unique challenges for elderly patients. Presence of age-related pharmacokinetic and pharmacodynamic changes, coupled with the high likelihood of comorbid conditions for which multiple medications are required, underscores the importance of monitoring the safe use of medication in this population [9]. To date, publications focused on managing C1-INH-HAE attacks in elderly patients are scarce. One observational registry study of 27 patients receiving an intravenously administered, plasma-derived C1-INH concentrate for the management of acute attacks demonstrated safe use in patients aged ≥ 65 years [10].

Icatibant, a subcutaneously administered antagonist of the bradykinin B_2_ receptor, has demonstrated efficacy and safety for the treatment of acute attacks in adults with HAE type I/II in three Phase 3 randomized, double-blind studies [11, 12]. However, mean patient age was < 65 years in all three trials [11, 12]. Although systemic exposure of icatibant has been shown to be increased in elderly patients, no effects on safety have been identified in this population [13].

The Icatibant Outcome Survey (IOS) is an ongoing international, prospective, observational drug registry study (NCT01034969) designed to monitor real-world safety and effectiveness of icatibant. Patients who are currently receiving, or are candidates for, treatment with icatibant are eligible to participate. Here, we compare patient characteristics and safety-related findings in elderly versus younger patients with C1-INH-HAE type I/II enrolled in the IOS.

## Methods

### Study design

Data for this analysis were collected from July 2009 through February 28, 2018, across 59 sites in 13 countries. The IOS study is conducted in compliance with the Declaration of Helsinki and the International Conference on Harmonisation Good Clinical Practice guidelines. Approvals were received from local ethics committees and/or health authorities, and patients provided written informed consent to participate.

Study design is presented in detail elsewhere [14]. Briefly, patient data were recorded via electronic forms by physicians at baseline and during follow-up visits (occurring approximately every 6 months), including frequency and severity of icatibant-treated attacks since the previous visit, safety/tolerability issues with icatibant treatment, presence of comorbid conditions and use of concomitant medications.

### Adverse event-related outcome measures

Adverse events (AEs) were assessed by frequency of occurrence, relationship to icatibant (possibly/probably/not related), and seriousness and severity (mild/moderate/severe). AEs classified as “possibly related to treatment” were those that occurred within a reasonable time sequence following administration of icatibant and are biologically plausible. Alternatively, the AE could be explained by concurrent disease or other drugs/chemicals. AEs classified as “probably related to treatment” were those that occurred within a reasonable time sequence following administration of icatibant, are biologically plausible, and are unlikely to occur as a result of concurrent disease or other drugs/chemicals.

AEs were categorized in accordance with the Medical Dictionary for Regulatory Activities system organ class and preferred term, and analyzed by the proportions of occurrence (percentage of patients reporting an event). For reporting seriousness/relationship of AEs to icatibant, each patient was counted only once, and only the highest seriousness/relationship category was reported for that patient.

### Statistical analysis

Descriptive analyses were conducted in younger patients (defined as ages < 65 years) versus elderly patients (≥ 65 years). Age at the time of data extract was used to define the age subgroups. For patients who discontinued early or died, age at discontinuation or death was used. Differences between age groups were evaluated using the Wilcoxon test for baseline characteristics; the Fisher test for possibly/probably related AEs and individual comparisons of mild, moderate, and severe AEs; and the Chi square test for individual comparisons of serious and non-serious AEs. Due to the observational nature of the registry, all analyses were considered exploratory; P-values were interpreted descriptively.

## Results

### Baseline demographics and disease characteristics

**Fig. 1:**
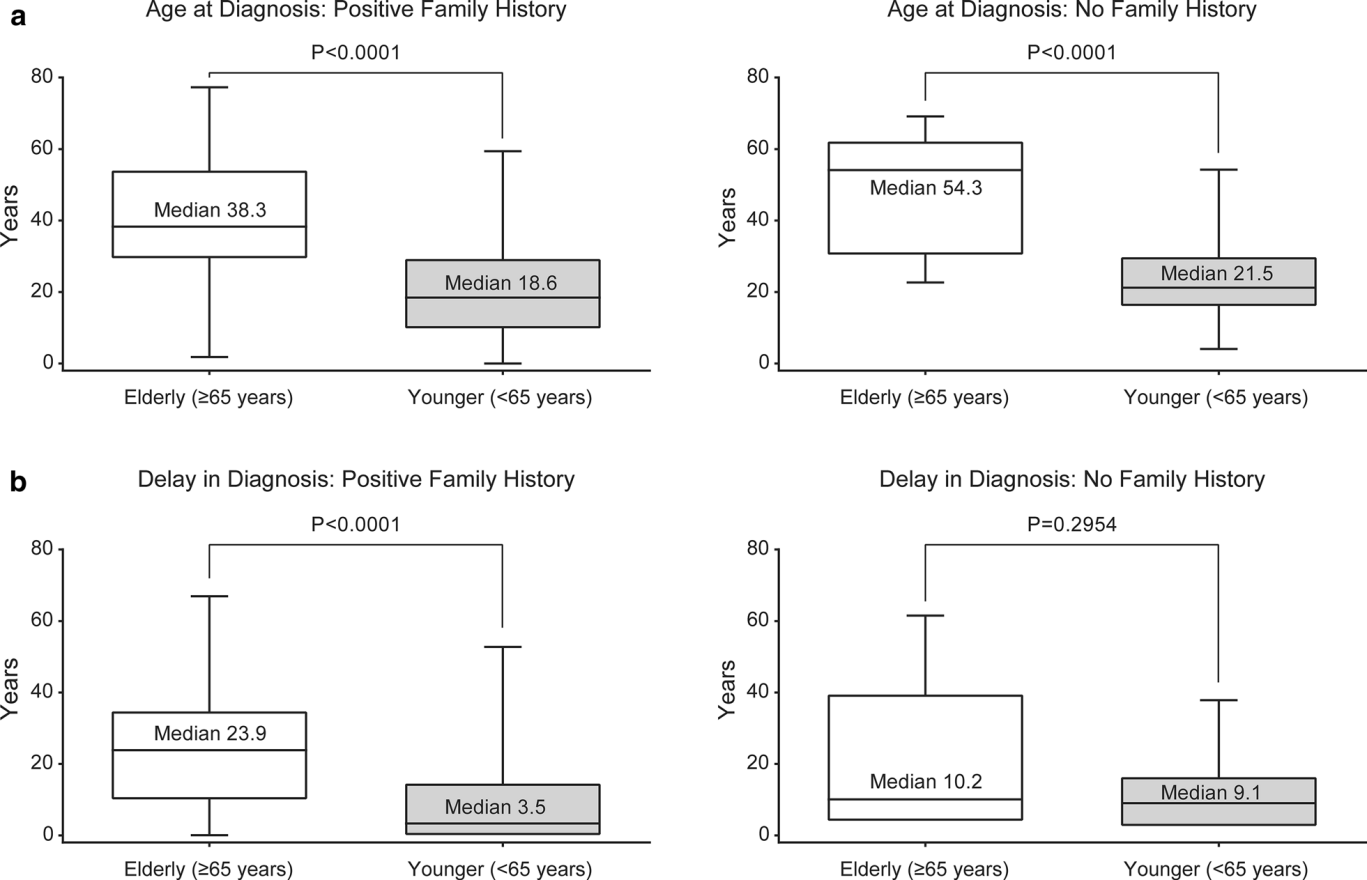
**a** Age at diagnosis in elderly and younger patients, with and without a family history and **b** delay in diagnosis in elderly and younger patients, with and without a family history^a^. The horizontal line inside each box indicates the median, the lower and upper borders of each box indicate the first and third quartiles, and the lowest and highest horizontal lines outside the box indicate the minimum and maximum value. ^a^A total of 113 patients with a family history had a negative delay in diagnosis (wherein diagnosis occurred before onset of symptoms, based on confirmatory laboratory testing); younger, n = 108; elderly, n = 5. Likewise, a total of five patients with no family history had a negative delay in diagnosis, all in the younger group

As of February 28, 2018, 872 patients with C1-INH-HAE type I/II were enrolled in the IOS (Table 1). Significant differences between elderly versus younger patients, respectively, were noted for age at symptom onset (median 17.0 vs 12.0 years, P = 0.0001), age at diagnosis (median 41.0 vs 19.4 years, P < 0.0001), and delay between symptom onset and diagnosis (median 23.9 vs 4.8 years, P < 0.0001 [Table 1]). When evaluated in relation to family history, median age at diagnosis was significantly higher in elderly than in younger patients, regardless of presence or absence of a family history (P < 0.0001, Fig. 1a). Whereas delay in diagnosis was significantly higher in elderly versus younger patients with a family history (23.9 vs 3.5 years, respectively, P < 0.0001); the difference between age groups was not significant in the absence of a family history (10.2 vs 9.1 years, P = 0.2954; Fig. 1b).

**Table 1:** Baseline characteristics

Characteristics	Elderly (≥ 65 years)	Younger (< 65 years)	Overall	P-value (elderly vs younger)
Patients, n (%)	100 (11.5)	772 (88.5)	872 (100)	
Type I	89 (89.0)	717 (92.9)	806 (92.4)	
Type II	11 (11.0)	55 (7.1)	66 (7.6)	0.1680
Sex				
Female, n (%)	51 (51.0)	471 (61.0)	522 (59.9)	0.0547
Age at enrollment (years)				
Median (Q1, Q3)	67.9 (63.8, 70.9)	36.8 (27.0, 47.3)	39.5 (27.9, 51.8)	< 0.0001
Age at symptom onset (years)				
Median (Q1, Q3)	17.0 (7.0, 25.0)	12.0 (5.0, 18.0)	12.0 (5.0, 19.0)	0.0001
Age at diagnosis (years)				
Median (Q1, Q3)	41.0 (30.8, 59.0)	19.4 (11.4, 30.2)	21.1 (13.1, 33.8)	< 0.0001
Delay in diagnosis, (years)				
Median (Q1, Q3)	23.9 (5.9, 37.2)	4.8 (0.21, 15.7)	6.1 (0.3, 17.5)	< 0.0001
Family history of C1-INH-HAE, n, %				
Yes	71 (71.0)	565 (73.2)	636 (72.9)	
No	11 (11.0)	100 (13.0)	111 (12.7)	
Unknown	7 (7.0)	50 (6.5)	57 (6.5)	
Missing	11 (11.0)	57 (7.4)	68 (7.8)	0.6051
Attack severity, n (%)				
Number of attacks^a^	581	5378	5959	
Very mild	11 (1.9)	18 (0.3)	29 (0.5)	
Mild	51 (8.8)	546 (10.2)	597 (10.0)	
Moderate	227 (39.1)	2202 (40.9)	2429 (40.8)	
Severe	219 (37.7)	1941 (36.1)	2160 (36.2)	
Very severe	73 (12.6)	671 (12.5)	744 (12.5)	
Very mild, mild, moderate vs severe/very severe				0.4393
Attack location, n (%)				
Number of attacks^a^	726	6016	6742	
Skin	323 (44.5)	2702 (44.9)	3025 (44.9)	0.958
Abdomen	369 (50.8)	3510 (58.3)	3879 (57.5)	0.438
Larynx	38 (5.2)	293 (4.9)	331 (4.9)	
Other	70 (9.6)	216 (3.6)	286 (4.2)	
Use of long-term prophylaxis, n (%)	2 (3.2)	8 (2.0)	–	0.6329
Number of cardiovascular medications, per patient				
Number of patients	49	66	115	
Median (Q1, Q3)	2.0 (1.0, 3.0)	1.0 (1.0, 2.0)	1.0 (1.0, 2.0)	0.0019

*C1-INH-HAE* hereditary angioedema with C1 inhibitor deficiency, *Q* quartile

^a^Attacks with severity/location data included; attacks with “missing” or “unknown” severity/location excluded

Of attacks with available severity data, there was no difference between groups (P = 0.4393) when comparing very mild/mild/moderate attacks versus severe/very severe attacks (Table 1). Attacks at a single site versus multiple sites comprised the majority of attacks in both the elderly and younger groups (elderly, 653/726 attacks [89.9%]; younger, 5339/6016 attacks [88.7%]) and there was no significant difference in attack location (Table 1).

### Comorbid conditions and their treatment

Elderly patients were significantly more likely than younger patients to have multiple comorbidities, both at baseline (IOS entry; 52.0% vs 11.1%, P < 0.0001) and during the follow-up period (26.0% vs 11.5%, P = 0.0002). For example, elderly patients were significantly more likely to have hypertension, ischemic heart disease, heart failure, stroke/transient ischemic attack, bleeding disorders, gastrointestinal disorders, diabetes, pulmonary disease, or renal-urogenital disorders at baseline, and hypertension, bleeding disorders, diabetes, and hepatic disorders during the follow-up period (Tables 2, 3). Similarly, elderly patients received a greater number of concomitant medications for cardiovascular/cerebrovascular conditions, both at baseline (median 2.0 vs 1.0 medications per patient, P = 0.0019; Table 1) and during the follow-up period (median 2.0 vs 1.0 medications per patient, P = 0.1238).

**Table 2:** Select comorbid conditions at baseline and follow-up

Comorbid conditions, n (%)	Baseline			Follow-up		
	Elderly (≥ 65 years)	Younger (< 65 years)	P	Elderly (≥ 65 years)	Younger (< 65 years)	P
Bleeding	4 (4.0)	5 (0.6)	0.0130	4 (4.0)	5 (0.6)	0.0130
Cardiovascular/cerebrovascular	50 (50)	69 (8.9)	< 0.0001	26 (26.0)	49 (6.3)	<0.0001
Diabetes	11 (11.0)	17 (2.2)	0.0001	6 (6.0)	8 (1.0)	0.0027
Gastrointestinal	11 (11.0)	19 (2.5)	0.0002	7 (7.0)	24 (3.1)	0.0757
Hepatic	1 (1.0)	0 (0.0)	0.1147	2 (2.0)	1 (0.1)	0.0362
Immunological	2 (2.0)	25 (3.2)	0.7592	2 (2.0)	36 (4.7)	0.3008
Infections	1 (1.0)	8 (1.0)	1.0000	4 (4.0)	25 (3.2)	0.5650
Neoplasia	3 (3.0)	6 (0.08)	0.0735	0 (0.0)	2 (0.3)	1.0000
Neurological	1 (1.0)	9 (1.2)	1.0000	3 (3.0)	9 (1.2)	0.1498
Osteoarticular	1 (1.0)	7 (0.9)	1.0000	2 (2.0)	17 (2.2)	1.0000
Psychiatric	7 (7.0)	23 (3.0)	0.0706	6 (6.0)	25 (3.2)	0.1549
Pulmonary	7 (7.0)	11 (1.4)	0.0023	2 (2.0)	10 (1.3)	0.6380
Renal/urogenital	4 (4.0)	5 (0.6)	0.0130	3 (3.0)	6 (0.8)	0.0735

**Table 3:** Cardiovascular comorbidities at baseline and follow-up

Comorbid conditions, n (%)	Baseline			Follow-up		
	Elderly (≥ 65 years)	Younger (< 65 years)	P	Elderly (≥ 65 years)	Younger (< 65 years)	P
Hypertension	48 (51.6)	64 (9.0)	< 0.0001	13 (18.1)	27 (5.3)	0.0004
Angina	2 (2.2)	4 (0.6)	0.1435	0 (0.0)	1 (0.2)	1.0000
Ischemic heart disease	5 (5.4)	5 (0.7)	0.0028	0 (0.0)	2 (0.4)	1.0000
Heart failure	2 (2.2)	1 (0.1)	0.0363	1 (1.4)	0 (0.0)	0.1248
Stroke/transient ischemic attack	6 (6.5)	5 (0.7)	0.0006	1 (1.4)	3 (0.6)	0.4147

### AEs related to icatibant treatment

During the time covered by this analysis, icatibant was used to treat 6798 attacks in 574 patients, with 715 icatibant-treated attacks reported by 63 (63.0%) elderly patients. Elderly and younger patients were similarly likely to have at least one treated attack (63.0% vs 66.2%, P = 0.527).

A similar percentage of elderly and younger patients experienced at least one AE possibly or probably related to icatibant (2.0% vs 2.7%; Table 4). No serious AEs (SAEs) occurring in elderly patients were judged to be possibly related to icatibant, whereas two younger patients reported three possibly related SAEs; one patient reporting gastritis (n = 1) and reflux esophagitis (n = 1), the other patient reporting angioedema crisis (n = 1).

**Table 4:** Overall summary of AEs

	Elderly (≥ 65 years)		Younger (< 65 years)	
	Patients (n = 100)	Events	Patients (n = 772)	Events
Total AEs, n (%)	28 (28.0)	86 (100.0)	164 (21.2)	374 (100.0)
Occurrence of ≥ 1 icatibant-related AE, n (%)	2 (2.0)	19 (22.1)	21 (2.7)	68 (18.2)
AEs by relationship to icatibant use, n (%)				
Not recorded	0 (0.0)	0 (0.0)	11 (1.4)	28 (7.5)
Not related	26 (26.0)	67 (77.9)	132 (17.1)	278 (74.3)
Possibly related	0 (0.0)	0 (0.0)	5 (0.6)	34 (9.1)
Probably related	2 (2.0)	19 (22.1)	16 (2.1)	34 (9.1)
Icatibant-related AEs by seriousness, n (%)				
Serious	0 (0.0)	0 (0.0)	2 (0.3)	3 (4.4)
Not serious	2 (2.0)	19 (100.0)	19 (2.5)	65 (95.6)

*AE* adverse event

The occurrence of mild and moderate AEs was similar between elderly and younger patients (P = 0.2170 and P = 0.2666, respectively). During the study, there were no deaths linked to treatment with icatibant.

A total of eight younger patients experiencing AEs had an older family member with C1-INH-HAE, however only two of these younger patients had an older family member (their father) who also experienced AEs. No patterns were found in the type of AEs experienced by related patients.

### Icatibant-related AEs by system organ class

Excluding off-label use and pregnancy-related AEs (evaluated for regulatory purposes), all related events in elderly patients were attributed to general disorders/administration site conditions, whereas treatment-related events in younger patients occurred across various system organ classes, including general disorders/administration site conditions, vascular disorders, skin/subcutaneous tissue disorders, gastrointestinal disorders, nervous system disorders, and others (Table 5).

**Table 5:** AEs possibly/probably related to icatibant, by system organ class and preferred term^a^

Patients	Elderly (≥ 65 years) (n = 100)	Younger (< 65 years) (n = 772)	Overall (n = 872)
Any AE n (%)	2 (2.0)	21 (2.7)	23 (2.6)
General disorders and administration site conditions	2 (2.0)	15 (1.9)	17 (1.9)
Injection site erythema	1 (1.0)	9 (1.2)	10 (1.1)
Pain	0 (0.0)	3 (0.4)	3 (0.3)
Application site erythema	0 (0.0)	2 (0.3)	2 (0.2)
Application site pain	0 (0.0)	2 (0.3)	2 (0.2)
Infusion site pain	1 (1.0)	1 (0.1)	2 (0.2)
Drug ineffective	0 (0.0)	1 (0.1)	1 (0.1)
Infusion site erythema	1 (1.0)	0 (0.0)	1 (0.1)
Injection site pain	1 (1.0)	0 (0.0)	1 (0.1)
Injection site hemorrhage	0 (0.0)	1 (0.1)	1 (0.1)
Localized edema	0 (0.0)	1 (0.1)	1 (0.1)
Non-cardiac chest pain	0 (0.0)	1 (0.1)	1 (0.1)
Edema	0 (0.0)	1 (0.1)	1 (0.1)
Therapeutic product ineffective	0 (0.0)	1 (0.1)	1 (0.1)
Infusion site urticaria	1 (1.0)	0 (0.0)	1 (0.1)
Administration site reaction	1 (1.0)	0 (0.0)	1 (0.1)
Asthenia	0 (0.0)	1 (0.1)	1 (0.1)
Vascular disorders	0 (0.0)	3 (0.4)	3 (0.3)
Hyperemia	0 (0.0)	2 (0.3)	2 (0.2)
Hot flush	0 (0.0)	1 (0.1)	1 (0.1)
Skin and subcutaneous tissue disorders	0 (0.0)	3 (0.4)	3 (0.3)
Skin reaction	0 (0.0)	2 (0.3)	2 (0.2)
Angioedema	0 (0.0)	1 (0.1)	1 (0.1)
Gastrointestinal disorders	0 (0.0)	4 (0.5)	4 (0.5)
Nausea	0 (0.0)	2 (0.3)	2 (0.2)
Abdominal distension	0 (0.0)	1 (0.1)	1 (0.1)
Abdominal pain upper	0 (0.0)	1 (0.1)	1 (0.1)
Reflux esophagitis	0 (0.0)	1 (0.1)	1 (0.1)
Hiatus hernia	0 (0.0)	1 (0.1)	1 (0.1)
Gastritis	0 (0.0)	1 (0.1)	1 (0.1)
Nervous system disorders	0 (0.0)	1 (0.1)	1 (0.1)
Post-herpetic neuralgia	0 (0.0)	1 (0.1)	1 (0.1)
Investigations	0 (0.0)	1 (0.1)	1 (0.1)
Blood pressure decreased	0 (0.0)	1 (0.1)	1 (0.1)
Infections and infestations	0 (0.0)	1 (0.1)	1 (0.1)
Herpes zoster	0 (0.0)	1 (0.1)	1 (0.1)
Psychiatric disorders	0 (0.0)	1 (0.1)	1 (0.1)
Depression	0 (0.0)	1 (0.1)	1 (0.1)

^a^Excludes AEs related to off-label use and pregnancy; *AE* adverse event

## Discussion and conclusions

To our knowledge, this is the first analysis that compares patient characteristics and safety and tolerability of icatibant in elderly versus younger patients with C1-INH-HAE. Findings from our analysis revealed a significantly older age at diagnosis in elderly versus younger patients with C1-INH-HAE, regardless of family history. Interestingly, delay in diagnosis was significantly longer in elderly versus younger patients in the presence of a positive family history, whereas the delay was similar for both age groups in the absence of a family history. This may have occurred due to the substantially larger number of younger patients with a positive family history (prompting earlier diagnosis), as well as better diagnostic work-up in families in current clinical practice. The delay in elderly patients with a family history may also be caused by the fact that their relatives from previous generations may have given up seeking medical advice for their condition and were living with symptoms, having lacked a formal diagnosis or effective C1-INH-HAE treatments in their own youth.

Our analysis did not reveal any new or unexpected safety concerns. Despite the fact that elderly patients had significantly more comorbidities and were receiving a greater number of concomitant medications, the occurrence of icatibant-related AEs was similar in the treated elderly versus younger population, with no significant differences noted between groups with regard to seriousness or severity of AEs. These findings correlate with those previously reported in an observational drug registry study by Bygum et al., who found no unexpected safety concerns in older versus younger patients treated with an intravenously administered, plasma-derived C1-INH in a real-world setting [10]. Additionally, no trends were noted with regard to the type of AEs experienced by younger patients who were related to an older patient enrolled in the IOS.

Our study had several limitations, including an uncontrolled clinical environment inherent in an observational study design; description of AEs relied heavily on patient recall. Additionally, substantially fewer elderly than younger patients were enrolled.

Despite the limitations, our real-world analysis provides valuable insight with regard to clinical characteristics of elderly patients with C1-INH-HAE and the safety of treating acute attacks with icatibant in this patient population.

## Data Availability

Shire provides access to the de-identified individual participant data (IPD) for eligible studies to aid qualified researchers in addressing legitimate scientific objectives. These IPDs will be provided following approval of a data sharing request, and under the terms of a data sharing agreement.

## Abbreviations

AE: adverse event
C1-INH-HAE: hereditary angioedema with C1 inhibitor deficiency
IOS: Icatibant Outcome Survey
SAE: serious adverse event
